# Sulphate of Quinine in Large Doses, in Typhoid Fever

**Published:** 1846-08

**Authors:** 


*Sulphate of Quinine in Large Doses, in Typhoid Fever.* M. Paul Boucher de la ville Jossy has undertaken a series of investigations on the physiological and therapeutical action of the sulphate of quinine in large doses, in typhoid fever—a subject which has recently attracted much attention among French practitioners. The following are the conclusions which he thinks himself justified in forming, as the result of his own personal observations:—

1. The non-acid sulphate of quinine, in the dose of from two to four grammes in a mixture of 125 grammes, administered in spoonfuls by the mouth, every two hours or more, does not produce any serious consequences.

2. It is generally taken with repugnance; often immediately after having been admitted into the stomach, producing a temporary nausea, and sometimes vomiting.

3. The mucous membrane of the digestive passages does not experience from it any injurious influence; there is only some slight sensation of heat in the course of the œsophagus to the cardia.

4. The eruption of the lenticular spots of the skin and sudamina is not modified, and it appears to be the same also with the intestinal eruption.

5. Its administration is often followed by a remarkable amendment, which is sometimes only transitory.

6. The apparent convalescence is generally rapid, but it is not the same with confirmed convalescence.

7. This apparent convalescence is owing to the modification of the general condition; the intestines not partaking in this modification.

8. The nervous phenomena and the slowness of the circulation, which are caused by the quinine, soon cease when the administration of the medicine is suspended.

9. It diminishes the head-ache, and often causes it to disappear; the pain is then replaced by heaviness of the head.

10. It often hastens the return of natural sleep.

11. Finally, it does not appear that the sulphate of quinine should constitute a special method of treatment, but it may prove serviceable combined with other means.—*Prov. Med. and Surg. Journ. from Gaz. Méd. de Paris.*

